# Supplementary material for: Reconstructing the sequence specificities of RNA-binding proteins across eukaryotes
Source: bioRxiv. 2024 Oct 18:2024.10.15.618476. Preprint. [Version 1] doi: 10.1101/2024.10.15.618476 (PMC11507768; doi:10.1101/2024.10.15.618476)
Supplement: 9 [file NIHPP2024.10.15.618476v1-supplement-9.pdf]

bioRxiv preprint doi: https://doi.org/10.1101/2024.10.15.618476; this version posted October 18, 2024. The copyright holder for this preprint (which was not certified by peer review) is the author/funder, who has granted bioRxiv a license to display the preprint in perpetuity. It is made available under aCC-BY 4.0 International license.

# Supplemental Figures

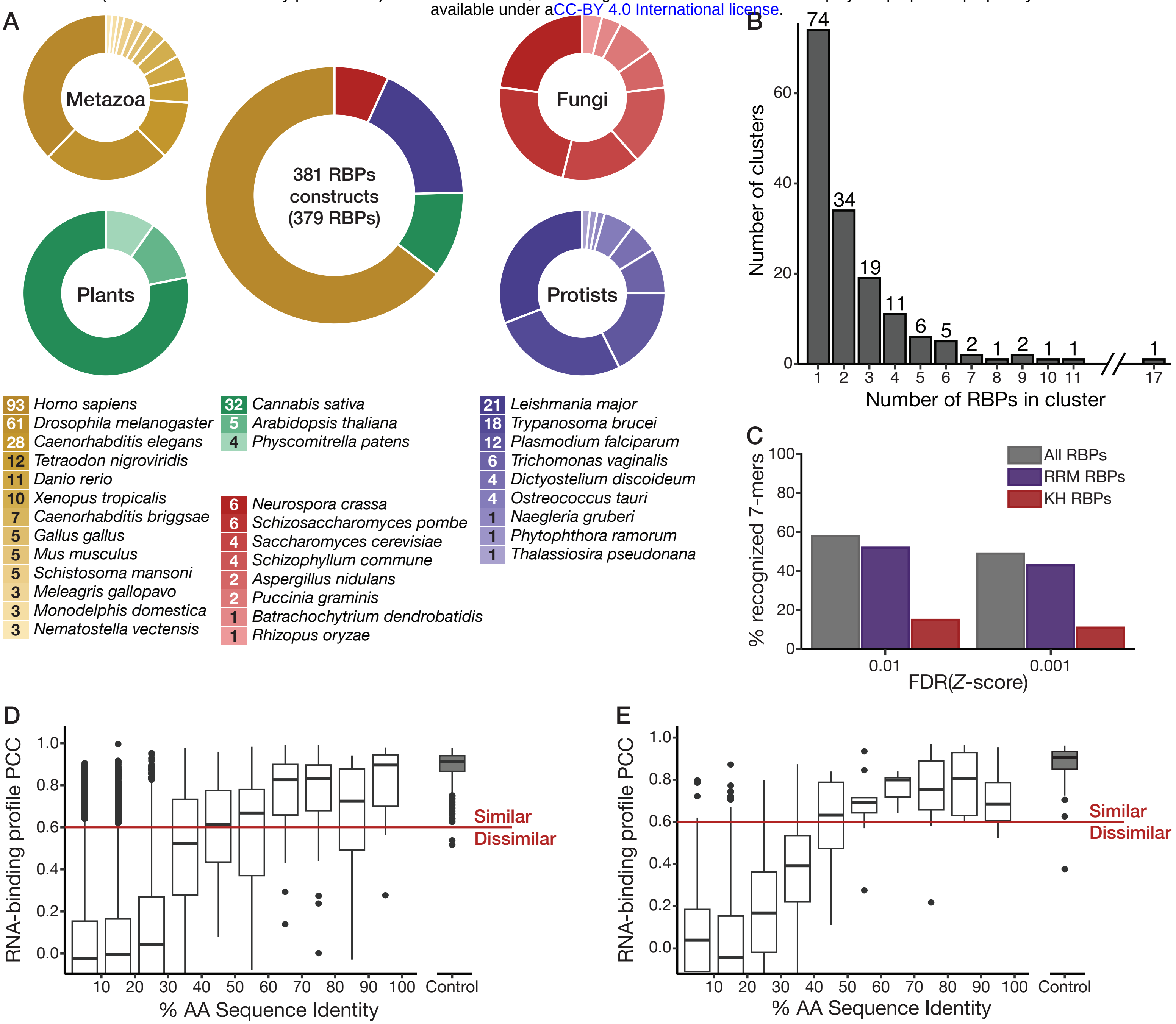

bioRxiv preprint doi: https://doi.org/10.1101/2024.10.15.618476; this version posted October 18, 2024. The copyright holder for this preprint (which was not certified by peer review) is the author/funder, who has granted bioRxiv a license to display the preprint in perpetuity. It is made available under aCC-BY 4.0 International license.

**Figure S1. RNAcompete-measured RNA sequence specificities.** **A**, The center donut chart depicts the breakdown of RNAcompete-measured RBPs across four eukaryotic kingdoms and 33 species, including both the RBPs measured for this study and those from Ray et al. 2013[1]. Clockwise from top left, donut charts depict the breakdown of RBPs by species for Metazoa, Fungi, Protists, and Plants. Legends adjacent to the donut charts show the number of measured RBPs for individual species. **B**, The RNAcompete-measured RBPs were split into clusters based on RNA-binding profile similarity; sequence specificities were hierarchically, agglomeratively clustered on the PCCs between RNA-binding profiles with complete linkage. Using a PCC cut-off of 0.6, 157 clusters were identified (**Table S1**) and the distribution of their sizes is displayed. **C**, Percentage of all 7-mers that are significantly bound (one-sided Z-test), FDR < 0.01 or < 0.001 (Benjamini-Hochberg FDR correction over the 16382 7-mers), by at least one RNAcompete-measured RBP, or at least one RNAcompete-measured RRM- or KH-domain RBP. **D**, **E**, Box plots show the distribution of RNA-binding profile PCCs for pairs of RBPs whose RBRs fall within the %AA ID range indicated on the x-axis. **D** depicts only the 308 RRM-domain-containing RBPs and **E** depicts only the 47 KH-domain-containing RBPs. As a control, the distribution of PCCs between RNAcompete Set A and Set B for the same experiment are displayed to the right.

bioRxiv preprint doi: https://doi.org/10.1101/2024.10.15.618476; this version posted October 18, 2024. The copyright holder for this preprint (which was not certified by peer review) is the author/funder, who has granted bioRxiv a license to display the preprint in perpetuity. It is made available under aCC-BY 4.0 International license.

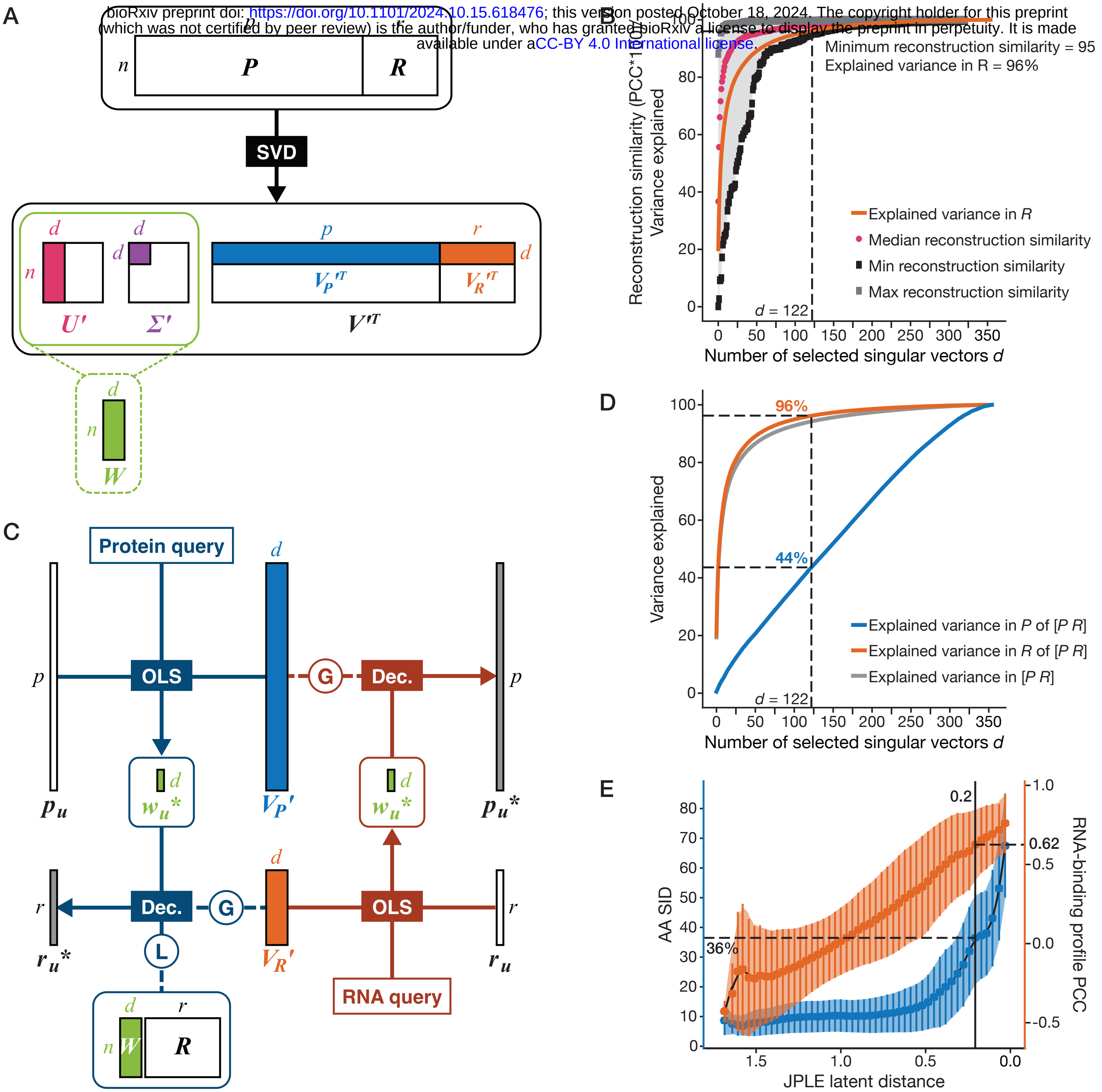

bioRxiv preprint doi: https://doi.org/10.1101/2024.10.15.618476; this version posted October 18, 2024. The copyright holder for this preprint (which was not certified by peer review) is the author/funder, who has granted bioRxiv a license to display the preprint in perpetuity. It is made available under aCC-BY 4.0 International license.

**Figure S2. JPLE captures the association between amino acid sequence and RNA sequence specificity. A,** Illustration of the JPLE training process for *n* RBPs. Singular value decomposition (SVD) is used to decompose the joint protein representation [***P R***] into ***U***, ***Σ***, and $\boldsymbol{V}^{T}$. The *d* singular vectors and values contributing the most to the variance of ***R*** in [***P R***] are selected, leading to the submatrices ***U'***, ***Σ'***, and $\boldsymbol{V'}^{T}$. The product ***W*** of ***U'*** and ***Σ'*** provides the *d*-dimensional latent embedding of the *n* RBPs. **B,** Distribution of the Pearson correlation coefficients (PCCs) between the reconstructed (***r****) and measured (***r***) RNA-binding profiles (i.e., the reconstruction similarity), as a function of the number of maintained singular vectors *d*. The orange line represents the total fraction of variance explained in ***R*** of [***P R***]. The median, minimal, and maximal reconstruction similarities are displayed and the distribution is indicated in gray. To enable a minimum reconstruction PCC of 0.95 for all measured RBPs, *d* = 122 is required. **C,** Illustration of the JPLE inference process for $RBP_u$. The left (in blue) showcases protein query, where the RBP's latent embedding $\boldsymbol{w_u}^*$ is obtained by deconvolving its peptide profile $\boldsymbol{p_u}$ into a mixture of the singular vectors in $\boldsymbol{V_P}'$. Its RNA-binding profile $\boldsymbol{r_u}^*$ could be reconstructed through either global (labeled G) or local (labeled L) decoding. The right (in brown) showcases RNA query, where the RBP's latent embedding $\boldsymbol{w_u}^*$ is obtained by deconvolving its RNA-binding profile $\boldsymbol{r_u}$ into a mixture of the singular vectors in $\boldsymbol{V_R}'$. Its peptide profile $\boldsymbol{p_u}^*$ could be reconstructed through global decoding. **D,** Variance explained in ***P*** and ***R*** of [***P R***], as a function of the number of selected singular vectors *d*. At *d* = 122, 44% and 96% of the total variance of ***P*** and ***R*** of [***P R***] are explained respectively. **E,** Relationship between RNA-binding profile PCCs and their JPLE latent distance (e-dist, i.e., cosine distance). JPLE was trained leaving clusters of RBPs with the same specificity (PCC>0.6) out, then embedding them into JPLE and measuring the distances between each other and to the RBPs in the training set. The latent distances between all RBP pairs were compared to their binding similarities (right y-axis) and the amino acid sequence identity (left y-axis). Lines and shaded area show smoothed mean and standard deviation across 50 equally sized bins. RBP pairs with < 0.20 latent distance possess an average RNA-binding similarity of 0.62 and AA SID of 37%.

bioRxiv preprint doi: https://doi.org/10.1101/2024.10.15.618476; this version posted October 18, 2024. The copyright holder for this preprint (which was not certified by peer review) is the author/funder, who has granted bioRxiv a license to display the preprint in perpetuity. It is made available under aCC-BY 4.0 International license.

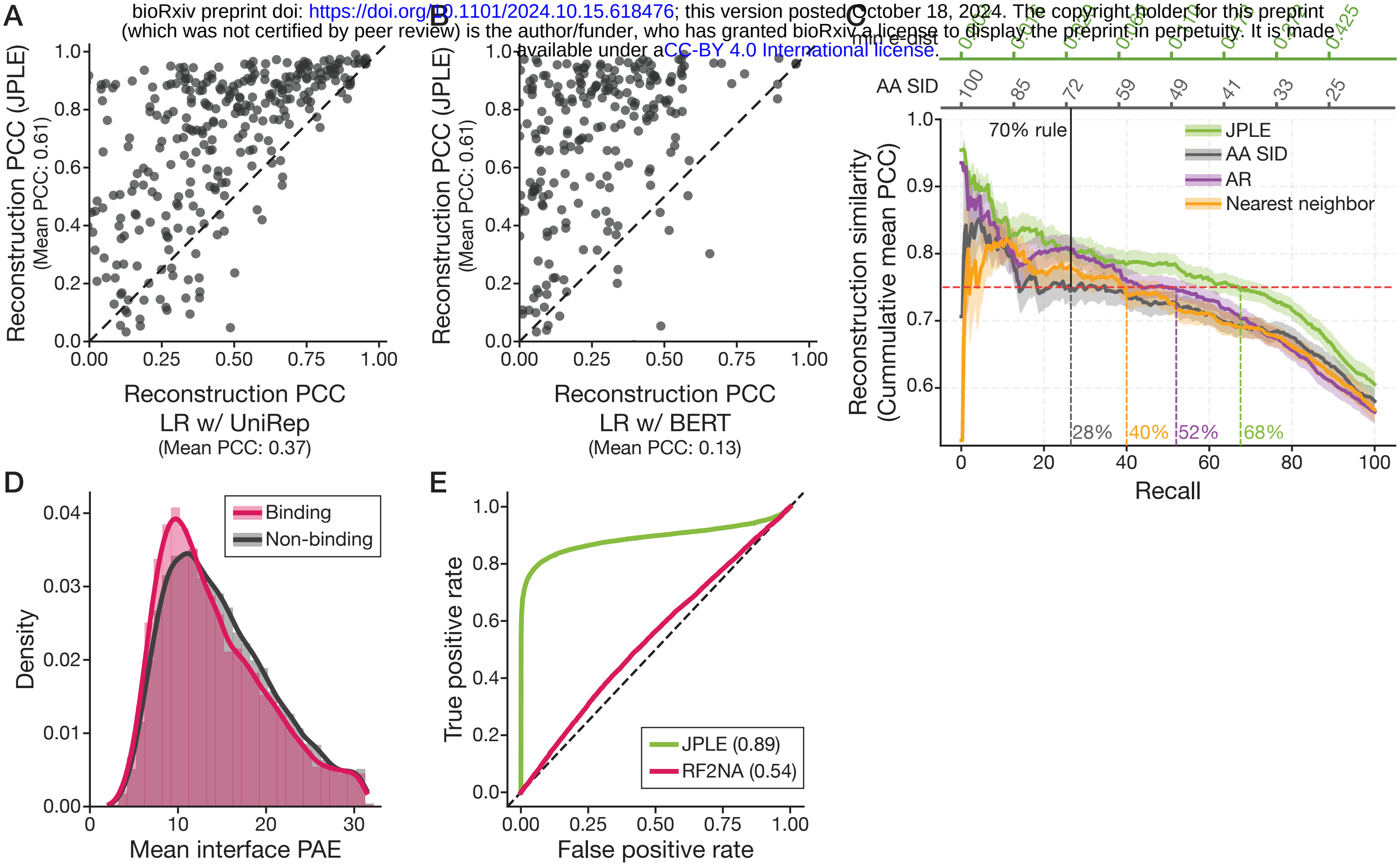

bioRxiv preprint doi: https://doi.org/10.1101/2024.10.15.618476; this version posted October 18, 2024. The copyright holder for this preprint (which was not certified by peer review) is the author/funder, who has granted bioRxiv a license to display the preprint in perpetuity. It is made available under aCC-BY 4.0 International license.

**Figure S3. JPLE outperforms alternative methods. A**, **B**, Comparison of the RNA-binding profile reconstructions generated by JPLE trained with 5-mer peptide features, to those generated by two Linear Regression (LR) models trained with deep learning features from TAPE: UniRep (**A**) and BERT (**B**). As in **Figure S2**, reconstruction PCCs are computed between the reconstructed (***r****) and measured (***r***) RNA-binding profiles. **C,** Precision-recall curves for RNA-binding profile reconstructions generated by JPLE, amino acid sequence identity (AA SID), Affinity Regression (AR), and the nearest neighbor model (see Methods). Precision (y-axis) is the mean PCC for reconstructions at least as confident as the threshold (top axes). JPLE confidence is e-dist to the nearest neighbor; AA SID confidence is % amino acid identity; AR confidence is one minus PCC between the test and nearest neighbor's embedding; the nearest neighbor model confidence is e-dist to the nearest neighbor. At the AA SID threshold of 70%, a mean PCC of 0.75 is achieved (red line). The recall for all four methods at a mean PCC of 0.75 is indicated. Standard error is shown in the shaded area around each line. **D**, Distribution of the mean interface predicted aligned errors (PAEs) for RoseTTAFold2NA's predicted structures with high-affinity 7-mers (binding) and low-affinity 7-mers (non-binding) for all 355 RBPs. **E**, ROC curves compare the performance of RoseTTAFold2NA and JPLE in the task of differentiating high-affinity from low-affinity 7-mers. The predictions for RoseTTAFold2NA and JPLE are the mean interface PAEs (see **D**) and the predicted Z-scores on held-out RBPs, respectively. Numbers in brackets indicate the AUROCs.

bioRxiv preprint doi: https://doi.org/10.1101/2024.10.15.618476; this version posted October 18, 2024. The copyright holder for this preprint (which was not certified by peer review) is the author/funder, who has granted bioRxiv a license to display the preprint in perpetuity. It is made available under aCC-BY 4.0 International license.

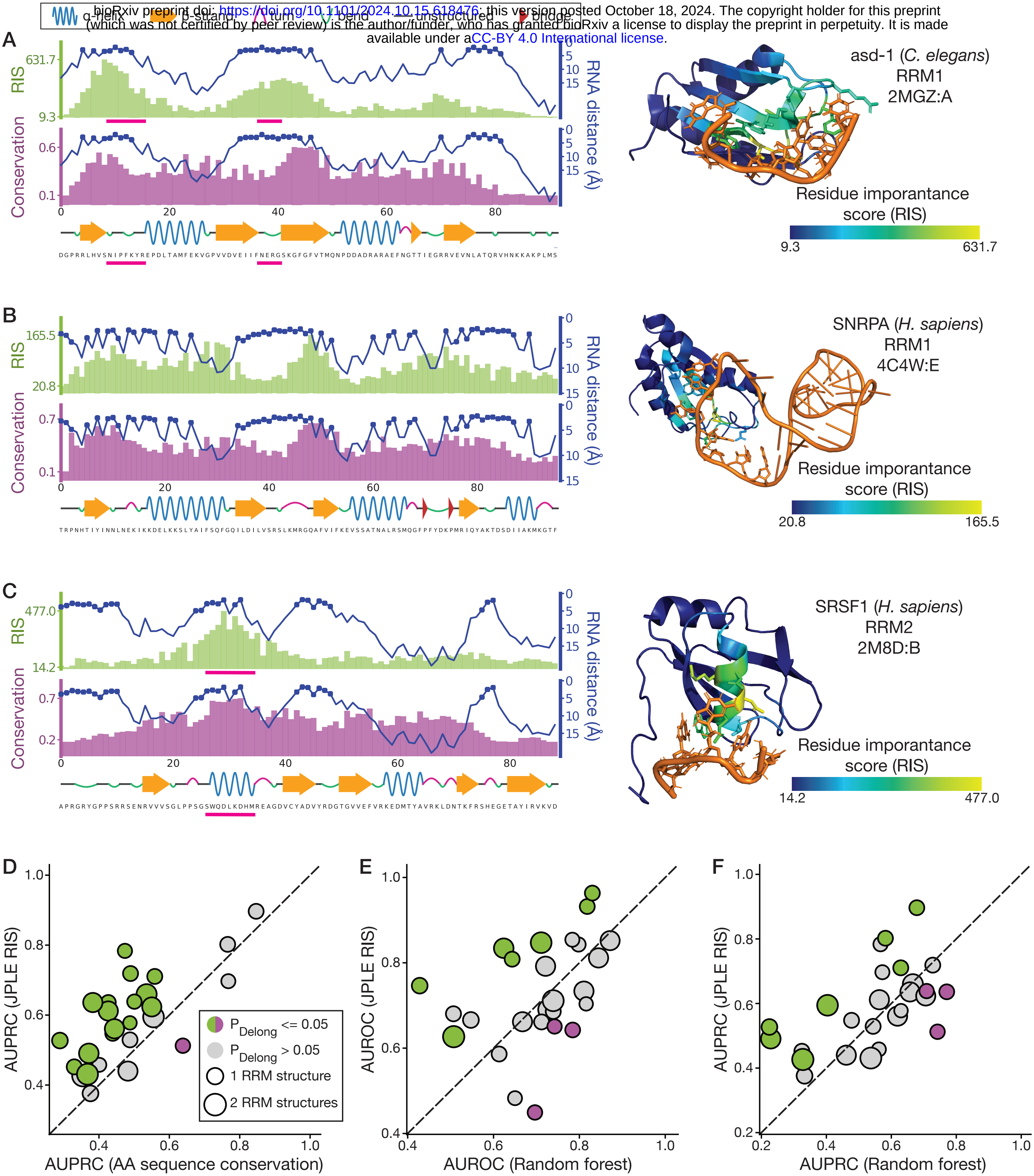

bioRxiv preprint doi: https://doi.org/10.1101/2024.10.15.618476; this version posted October 18, 2024. The copyright holder for this preprint (which was not certified by peer review) is the author/funder, who has granted bioRxiv a license to display the preprint in perpetuity. It is made available under aCC-BY 4.0 International license.

**Figure S4. JPLE predicts RNA-interacting amino acids. A-C**, The distance between individual residues and RNA (in Angstroms) is compared to JPLE residue importance scores (RISs) (top left) and conservation scores (middle left) for the RRM-RNA co-complex structure depicted to the right. A linear visualization of the protein secondary structure is depicted at the bottom left along with the protein sequence. RRM-RNA co-complex structures are coloured by JPLE RIS. The loops between β1 and α1 and between β2 and β3 found to confer specificity in RBFOX1[2], the human homolog of *C. elegans* ASD-1, are indicated in **A** with a pink bar below the amino acid sequence and below the RIS histogram. Similarly indicated in **C** is the α-helix that confers sequence specificity in the depicted SRSF1 RRM[3]. **D**, Comparison between sequence conservation and JPLE RISs for predicting RRM domain interface residues, evaluated with AUPRC. Coloured circles indicate a significant difference in performance between the two scoring methods, as determined by the Delong test using AUROC values as in **Figure 3C**. **E-F**, Comparison between JPLE RIS and a random forest model trained using the following features: amino acid position specific sequence matrices from multiple sequence alignment, physico-chemical residue features, residue identity, conservation within a window of five amino acids. Results of evaluation by AUROC are shown in **E**, and by AUPRC in **F**. Points in both panels are coloured according to significance of the Delong test performed on AUROC values.

bioRxiv preprint doi: https://doi.org/10.1101/2024.10.15.618476; this version posted October 18, 2024. The copyright holder for this preprint (which was not certified by peer review) is the author/funder, who has granted bioRxiv a license to display the preprint in perpetuity. It is made available under aCC-BY 4.0 International license.

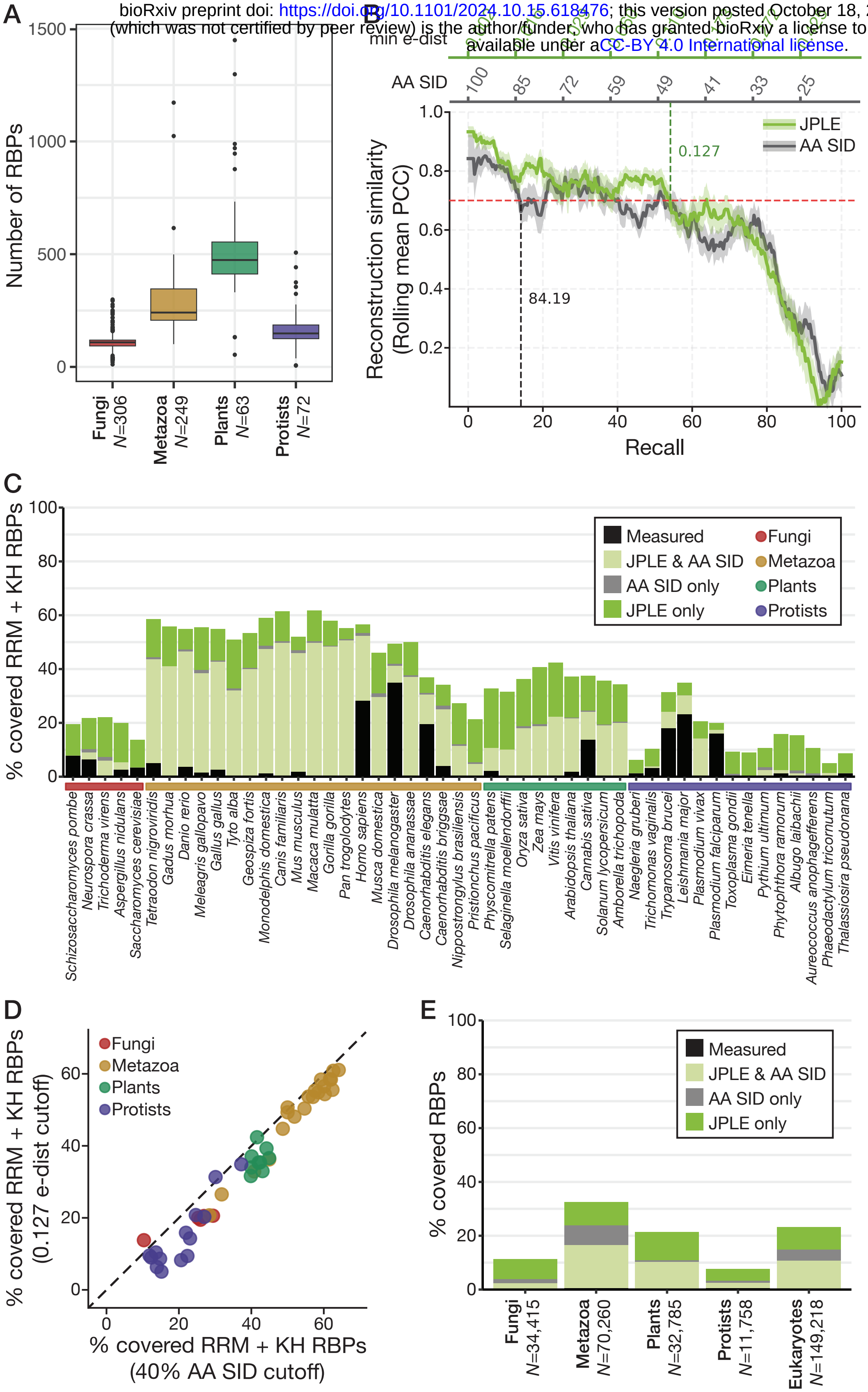

bioRxiv preprint doi: https://doi.org/10.1101/2024.10.15.618476; this version posted October 18, 2024. The copyright holder for this preprint (which was not certified by peer review) is the author/funder, who has granted bioRxiv a license to display the preprint in perpetuity. It is made available under aCC-BY 4.0 International license.

**Figure S5. JPLE reconstructs RNA-binding specificities for thousands of eukaryotic RBPs.** **A,** The number of RBPs identified in each of 690 eukaryotes split across four kingdoms. **B,** Precision-recall curves for RNA-binding profile reconstructions generated by amino acid sequence identity (AA SID) and JPLE. Precision (y-axis) is the mean rolling Pearson Correlation Coefficient (PCC) for reconstructions at least as confident as the threshold (top axes). The selection size for the rolling average window is 25 reconstructions. AA SID confidence is % amino acid identity, JPLE confidence is the minimum e-dist. Grey and green dashed lines indicate the confidence at which the rolling mean PCC first hits 0.70 (red dashed line). Standard error is shown in the shaded area around each line. **C,** The fraction of measured and reconstructed specificities for RRM- and KH-domain-containing RBPs for 49 representative species. The proportion of reconstructed specificities that were identified by AA SID, JPLE, or both are indicated. The kingdom to which the species belongs is indicated below the x-axis. **D**, Scatterplot displays the percentage of specificities for RRM- and KH-domain-containing RBPs that were reconstructed by JPLE (with an e-dist cutoff of 0.127) compared to a 40% AA SID for 49 representative species (listed in panel **C**). **E**, The fraction of measured and reconstructed RBP specificities for four eukaryotic kingdoms and all eukaryotes contained in EuPRI and on CisBP-RNA. This plot includes measured and reconstructed RBPs that do not contain RRM- or KH-domain-containing RBPs.

bioRxiv preprint doi: https://doi.org/10.1101/2024.10.15.618476; this version posted October 18, 2024. The copyright holder for this preprint (which was not certified by peer review) is the author/funder, who has granted bioRxiv a license to display the preprint in perpetuity. It is made available under aCC-BY 4.0 International license.

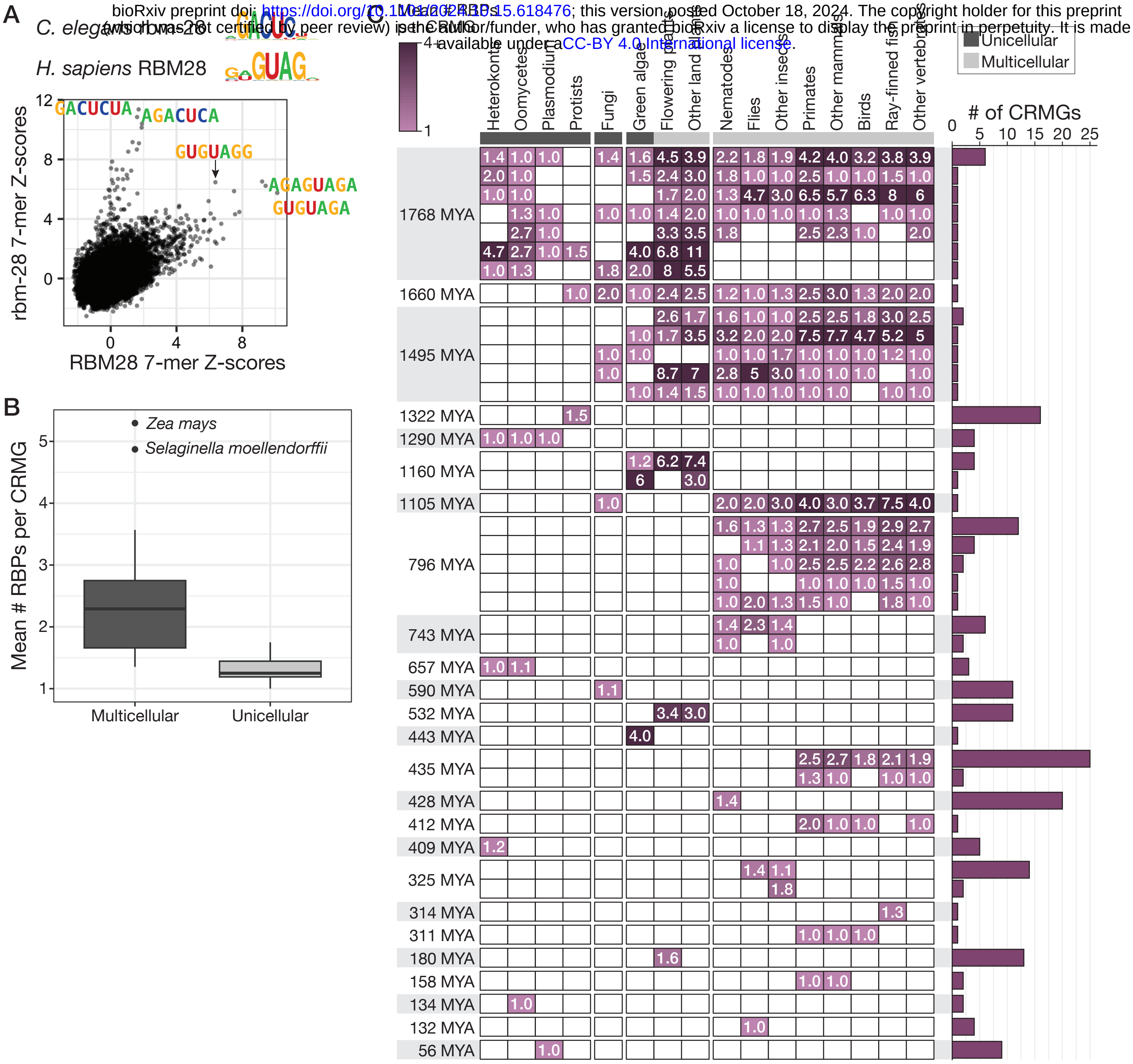

bioRxiv preprint doi: https://doi.org/10.1101/2024.10.15.618476; this version posted October 18, 2024. The copyright holder for this preprint (which was not certified by peer review) is the author/funder, who has granted bioRxiv a license to display the preprint in perpetuity. It is made available under aCC-BY 4.0 International license.

**Figure S6. Evolution of eukaryotic CRMGs. A**, RNAcompete 7-mer Z-scores are compared between human RBM28 and *C. elegans* ortholog *rbm-28*. RNAcompete motifs are shown above the plot, and some top 7-mers are directly labelled. **B**, The mean number of RBPs contained within a CRMG for multicellular (N=36) and unicellular (N=17) species. Only multi-species CRMGs containing an RBP with an e-dist of <0.2 to an RNAcompete-measured RBP were used for the calculations. Outlier species are labelled. **C**, The number of gained CRMGs at different time points are broken down by the major eukaryotic clades to which they belong. Cells in the heatmap are darkened to indicate the presence, in a given clade, of the CRMGs in the barplot to the right. The mean number of RBPs within the associated set of CRMGs in extant species belonging to the associated clade is displayed in each cell. The barplot on the right displays the number of CRMGs shared between the indicated clades that arose at the given time point. As in **B**, only multi-species CRMGs containing an RBP with an e-dist of <0.2 to an RNAcompete-measured RBP are shown.

bioRxiv preprint doi: https://doi.org/10.1101/2024.10.15.618476; this version posted October 18, 2024. The copyright holder for this preprint (which was not certified by peer review) is the author/funder, who has granted bioRxiv a license to display the preprint in perpetuity. It is made available under aCC-BY 4.0 International license.

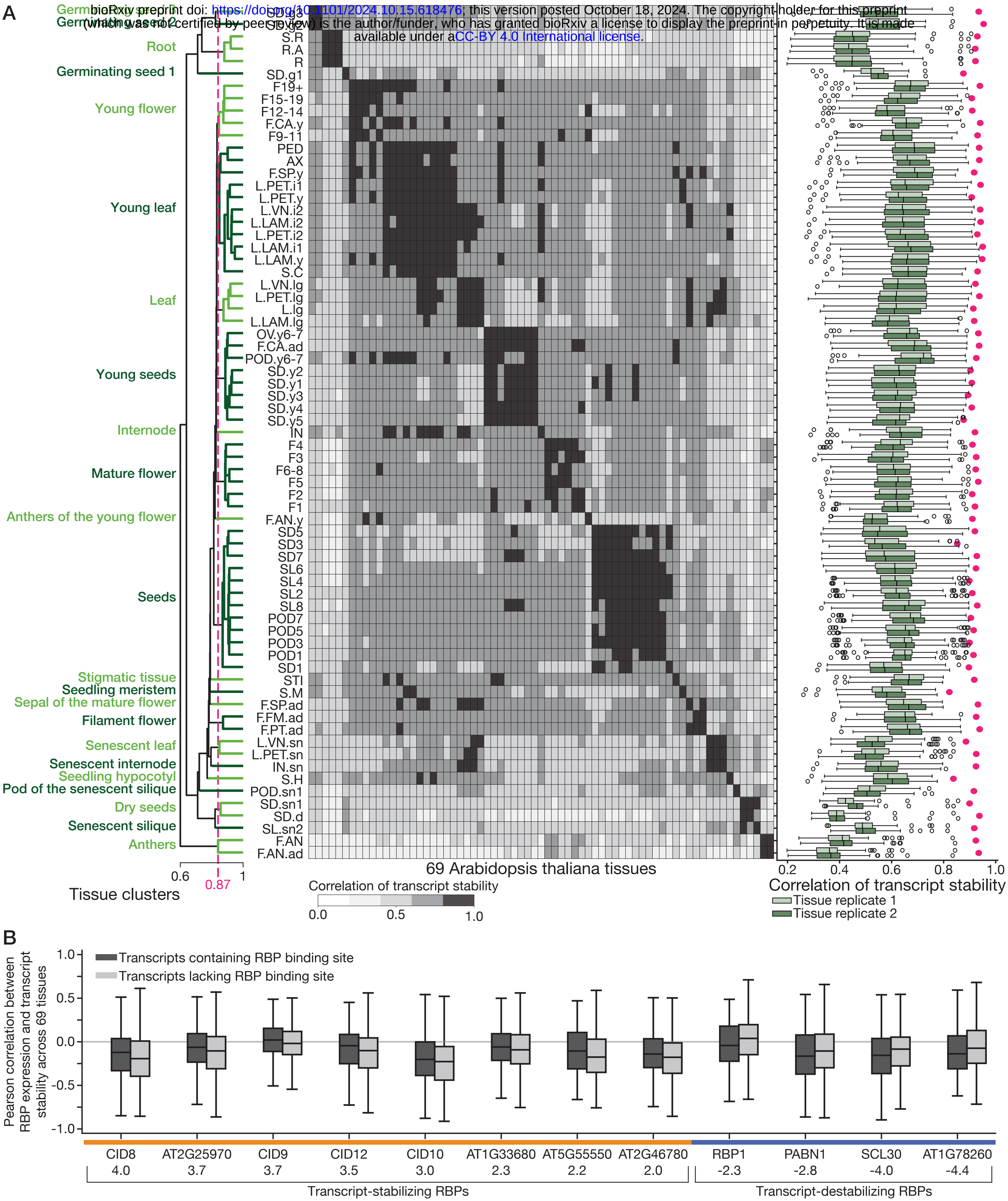

bioRxiv preprint doi: https://doi.org/10.1101/2024.10.15.618476; this version posted October 18, 2024. The copyright holder for this preprint (which was not certified by peer review) is the author/funder, who has granted bioRxiv a license to display the preprint in perpetuity. It is made available under aCC-BY 4.0 International license.

**Figure S7. Predicting RNA stability regulators with reconstructed RNA sequence specificities in Arabidopsis thaliana. A,** Heatmap displays the results of agglomerative clustering with single linkage on the Pearson Correlation Coefficients (PCCs) between the mRNA stability scores of the 6794 genes with reproducible scores. Tissue clusters were identified at a PCC cutoff of 0.87, generating 23 clusters, the names of which are indicated to the left and correspond to **Figure 6B**. To the right of the heatmap, boxplots display the mRNA stability score PCCs between each tissue replicate and all other tissue replicates, excluding the replicate from the same tissue. Tissue replicate correlations are displayed by the pink circle between each pair of boxes. **B,** Boxplot shows the distribution of PCCs between mRNA stability scores and RBP expression for the 12 RBPs identified as having a putative role in regulating stability.

bioRxiv preprint doi: https://doi.org/10.1101/2024.10.15.618476; this version posted October 18, 2024. The copyright holder for this preprint (which was not certified by peer review) is the author/funder, who has granted bioRxiv a license to display the preprint in perpetuity. It is made available under aCC-BY 4.0 International license.

## References


1 Ray, D. *et al.* A compendium of RNA-binding motifs for decoding gene regulation. *Nature* **499**, 172-177, doi:10.1038/nature12311 (2013).

2 Auweter, S. D. *et al.* Molecular basis of RNA recognition by the human alternative splicing factor Fox-1. *EMBO J* **25**, 163-173, doi:10.1038/sj.emboj.7600918 (2006).

3 Clery, A. *et al.* Isolated pseudo-RNA-recognition motifs of SR proteins can regulate splicing using a noncanonical mode of RNA recognition. *Proc Natl Acad Sci U S A* **110**, E2802-2811, doi:10.1073/pnas.1303445110 (2013).
